# Multisensory Integration as per Technological Advances: A Review

**DOI:** 10.3389/fnins.2021.652611

**Published:** 2021-06-22

**Authors:** Patricia Cornelio, Carlos Velasco, Marianna Obrist

**Affiliations:** ^1^Department of Computer Science, University College London, London, United Kingdom; ^2^Centre for Multisensory Marketing, Department of Marketing, BI Norwegian Business School, Oslo, Norway

**Keywords:** multisensory integration, human–computer interaction, multisensory technology, interaction techniques, sensory stimulation

## Abstract

Multisensory integration research has allowed us to better understand how humans integrate sensory information to produce a unitary experience of the external world. However, this field is often challenged by the limited ability to deliver and control sensory stimuli, especially when going beyond audio–visual events and outside laboratory settings. In this review, we examine the scope and challenges of new technology in the study of multisensory integration in a world that is increasingly characterized as a fusion of physical and digital/virtual events. We discuss multisensory integration research through the lens of novel multisensory technologies and, thus, bring research in human–computer interaction, experimental psychology, and neuroscience closer together. Today, for instance, displays have become volumetric so that visual content is no longer limited to 2D screens, new haptic devices enable tactile stimulation without physical contact, olfactory interfaces provide users with smells precisely synchronized with events in virtual environments, and novel gustatory interfaces enable taste perception through levitating stimuli. These technological advances offer new ways to control and deliver sensory stimulation for multisensory integration research beyond traditional laboratory settings and open up new experimentations in naturally occurring events in everyday life experiences. Our review then summarizes these multisensory technologies and discusses initial insights to introduce a bridge between the disciplines in order to advance the study of multisensory integration.

## Introduction

We perceive the world through multiple senses by collecting different sensory cues that are integrated or segregated in our brain to interact with our environment (Shams and Beierholm, 2011). Integrating information across the senses is key to perception and action and influences a wide range of behavioral outcomes, including detection (Lovelace et al., 2003), localization (Nelson et al., 1998), and, more broadly, reaction times (Diederich and Colonius, 2004; Stein, 2012). Advancing the study of multisensory integration helps us to understand the organization of sensory systems, and in applied contexts, to conceive markers (based on deficits in integration) of disorders, such as autism spectrum disorder (Feldman et al., 2018) and schizophrenia (Williams et al., 2010). This, in turn, demonstrates the importance of assessing and quantifying multisensory integration (Stevenson et al., 2014).

Many studies have been conducted to quantify multisensory integration. However, different challenges are highlighted in the literature (Stein et al., 2009; Stevenson et al., 2014; Colonius and Diederich, 2017, 2020). One of the most notable challenges is the need to control timing, spatial location, and sensory quality and quantity during stimulus delivery (Spence et al., 2001). Another challenge is the complexity of studying integration involving the chemical senses (smell and taste). Many studies typically rely on audio–visual interactions (Stein, 2012; Noel et al., 2018) because, among other reasons, the technology to deliver audio–visual stimuli is relatively well-established and widely available (e.g., screens, headphones). Emerging multisensory technologies from computer science, engineering, and human–computer interaction (HCI^1^) enable new ways to stimulate, replicate, and control sensory signals (touch, taste, and smell). Therefore, they could expand the possibilities for multisensory integration research. However, due to their recent emergence and rapid development, their potential to do so might be overlooked or underexplored.

**FIGURE 1:**
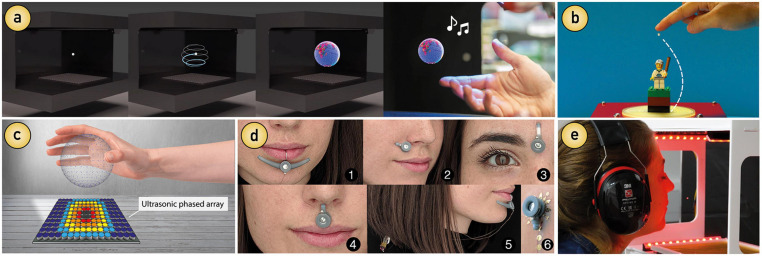
Emerging multisensory technologies exemplifying interface and device advancements. **(a)** Particle-based volumetric displays (Hirayama et al., 2019). **(b)** Acoustic metamaterials that bend the sound (Norasikin et al., 2018). **(c)** Mid-air haptic 3D shapes produced by focused ultrasound (Carter et al., 2013). **(d)** On-face olfactory interfaces (Wang et al., 2020). **(e)** Gustatory experiences based on levitated food (Vi et al., 2020).

For example, as shown in Figure 1, acoustic levitation techniques are employed to display visual content (that can be also heard and felt), addressing the common limitations of 2D screens and stereoscopic displays typically employed to deliver visual stimuli (Figure 1a). Acoustic metamaterials are used to “bend” the sound so that auditory stimuli can be directed from a static source to a specific location while, at the same time, providing tactile sensations (Figure 1b). Moreover, it is now also possible to control and deliver tactile sensations to the skin without the need of additional attachments (e.g., gloves or physical actuators) using focused ultrasound (Figure 1c). With regards to smell and taste stimulation, we are seeing growing development efforts to create more flexible and portable solutions that vary in their capabilities compared to established laboratory equipment, such as gustatometers and olfactometers. Importantly, emerging olfactory displays and smell-delivery technologies are becoming smaller, wearable, and more modular, enabling less invasive stimulation within and outside laboratory settings (Figure 1d). Similarly, we can see novel gustatory stimulation approaches emerging, such as taste levitation systems that exploit the principles of acoustics for delivering precisely controllable taste stimuli to the user’s tongue (Figure 1e).

In this review, we discuss the potential of these new and emerging multisensory technologies to expand the study of multisensory integration by examining opportunities to facilitate the control and manipulation of sensory stimuli beyond traditional methods and paradigms. The set of novel digital interfaces and devices that we review exemplifies technological advances in multisensory stimulation and their associated opportunities and limitations for research on multisensory integration. The ultimate aim of this review is to introduce a bridge between disciplines and encourage future development and collaboration between the engineers developing the technologies and scientists from psychology and neuroscience studying multisensory integration.

We close our review with a reflection on the growing multisensory human–computer integration^2^ symbiosis—when technology becomes an integral part of everyday life and activities. This fast-growing integration raises a range of ethical questions and considerations regarding shared responsibility between humans and systems. One highly important question is related to the sense of agency (SoA), often referred to as the feeling of being in control (Haggard, 2017). We live in an increasingly digital world in which intelligent algorithms (e.g., autonomous systems and autocomplete predictors) assist us and influence our behavior. We are therefore not always aware of the extent to which technology makes decisions for us, which raises the question, who is in control now? While emerging technology can provide further multisensory signals to promote a SoA (I am, who is acting), further discussion is needed in light of the rapid development of artificial intelligence systems. This review also aims to promote further discussion and reflection upon the role of the SoA and other relevant questions that emerge through the relationship between the senses and technology (Velasco and Obrist, 2020).

## Expanding Multisensory Integration: Current Tools/Methods and Emerging Technology

Studying how multiple sources of sensory information are integrated into a unified percept, often referred to as the “unity assumption” (Chen and Spence, 2017), has been a subject of intense research for many years. Studies employ different perspectives to explore multisensory integration. For example, some use weighted linear combination theories consisting of linear sums of unimodal sensory signals, wherein certain sensory modalities become more dominant than others to produce a unified perception (Ernst and Banks, 2002; Ernst and Bülthoff, 2004). Others explore sensory integration at the level of a single neuron (Stanford and Stein, 2007; Stein and Stanford, 2008) and explain the integration of sensory information through neural circuitry.

One common aspect to the study of multisensory integration is the need for a carefully controlled stimulus delivery. Computational and psychophysical studies must precisely present subjects with multisensory cues that have carefully controlled properties. Many such studies build on the modulation of the reliability of sensory cues to weigh the influence of individual sensory modalities (Ernst and Banks, 2002; Fetsch et al., 2012). For example, in a visuo–tactile task (e.g., size estimation of an object), I rely on my vision and touch to estimate the size of the object. Then, to examine how both of my senses are integrated, the researchers modify the reliability of the sensory information I perceived from the object. They might alter the clarity of my vision (e.g., by means of a special screen) or my perceived object size (e.g., by means of a shape-changing object). This modulation implies changing and varying the stimulus properties (Burns and Blohm, 2010; Parise and Ernst, 2017) requiring precise computer-controlled delivery. However, it has been suggested that many behavioral studies on multisensory integration rely on “century-old measures of behavior—task accuracy and latency” (Razavi et al., 2020) and are commonly constrained by in-laboratory and desktop-based settings (Wrzus and Mehl, 2015).

In the following sections, we present an overview of current challenges that can be overcome in light of new and emerging multisensory technologies. We particularly focus on technologies that illustrate the kinds of novel devices and methods emerging from HCI, which provide new functionalities for studying the human senses and which have not been used in multisensory integration research, although they can be of great interest and help in such research. Accordingly, we review novel, recently emerging technology that (1) is claimed to be multisensory/multimodal, (2) can be easily integrated with other multisensory technology, (3) allows naturalistic environments beyond laboratory settings, or (4) enables a move from physical to digital interactions.

We live in a time in which technology is ubiquitous, which means that delivering, measuring, and assessing multisensory signals in daily life can be facilitated. We have selected these particular technologies to highlight their potential to advance the study of multisensory integration not only by offering precision and controllability but also by enabling more natural study environments beyond desktop-based experiments. With this focus, we aim to examine opportunities that permit studies to take place over time (e.g., longitudinal studies) or outside a laboratory (e.g., at home) while still being precise. In the following sections, we describe the representative technological advancements for each of the main senses: vision, audition, touch, olfaction, and gustation (summarized in Table 1). We present separated sections for each of those senses to give focused information to readers with a particular interest (e.g., researchers interested in new olfactory technologies). In each section, we first introduce the emerging technology and benefits for individual sensory modalities, we then discuss and exemplify how it can aid multisensory integration research, and we further highlight how they can be integrated dynamically into multisensory paradigms, i.e., by capitalizing on the different technologies as modules to conduct studies involving multiple senses.

**TABLE 1:** Key properties of emerging multisensory technologies for each sensory modality.

**Sensory modality**	**Emerging technology**	**Key stimulation opportunities**	**Multisensory integration flexibility**	**Main advantages for multisensory experiments**	**Primary reference**
Vision	Volumetric displays	Depth	Visual content can be heard and felt simultaneously	No need of head-mounted displays while giving 3D cues in the real world	Hirayama et al., 2019
Audition	Acoustic lenses and metamaterials	Directionality	Audio signals can be felt and used to levitate and direct objects that create visual displays	Enable directional sound stimulation while giving freedom to navigate	Norasikin et al., 2018, 2019
Touch	Focused ultrasound	Un-instrumented 3D tactile sensations	The sound waves used to produce tactile sensation can also be heard and easily integrated into visual paradigms (e.g., virtual reality)	No need of physical actuators; open opportunities to introduce the study of integration of mid-air touch with other senses	Carter et al., 2013; Martinez Plasencia et al., 2020
Smell	Wearable smell technology	Portable and body-responsive	Its portability makes it easily integrated into other multisensory technologies	Delivery on-demand outside a laboratory setting, enabling daily life testing and longitudinal studies	Amores et al., 2018; Wang et al., 2020
Taste	Levitated food	Sterile, un-instrumented	Integration of levitated food with visual, olfactory, auditory, and tactile stimuli	Delivery actual food (multiple morsels) simultaneously in 3D, enabling the manipulation of food’s trajectories	Vi et al., 2017b, 2020

### Visual Stimulation Beyond the Screen

In the well-studied audio–visual integration space, visual information is modulated by altering the frequency or localization of seen and heard stimuli (Rohe and Noppeney, 2018), often by employing the established McGurk paradigm (Gentilucci and Cattaneo, 2005). In another example, for visuo–haptic integration studies, visual information is modulated in size estimation or identification tasks through the manipulation of an object’s physical shape (Yalachkov et al., 2015) or the alteration of digital images through augmented (Rosa et al., 2016) and virtual reality (VR) headsets (Noccaro et al., 2020).

For these studies, visual stimulus presentation is typically limited to 2D screens that show visual cues (static or in movement) in a two-dimensional space. While high-frequency 2D screens offer a good image presentation quality and low latency, they are still limited to 2D content, thus constraining depth perception. The stereoscopic displays used in VR headsets offer great advantages for 3D content visualization and full-body immersion also allowing the study of visuo-vestibular and proprioceptive signals (Gallagher and Ferrè, 2018; Kim et al., 2020) and even visuo–gustatory interactions (Huang et al., 2019). However, it is suggested that stereoscopic displays typically used in VR have disadvantages for psychology experiments. For example, people tend to consistently underestimate the size of the environment and their distance to objects (Wilson and Soranzo, 2015) even when motion parallax and stereoscopic depth cues are provided to the observer (Piryankova et al., 2013). This can be limiting for spatial tasks (e.g., in visuo–tactile interactions). Additionally, immersion in VR can cause cybersickness due to the brain receiving conflicting signals about the user position and its relation to the movement observed in the virtual environment (Gallagher and Ferrè, 2018).

The aforesaid challenges could be overcome through novel visual display technologies, such as advances in particle-based volumetric displays (PBDs) (Smalley et al., 2018). These displays provide a benefit over traditional 2D screens since they are not limited by two-dimensional content. PBDs show 3D images in mid-air, thus allowing depth perception, which could be integrated into traditional experimental paradigms, such as depth discrimination tasks (Deneve and Pouget, 2004; Rosa et al., 2016). Furthermore, PBDs also offer a benefit over VR headsets as these novel displays do not require wearing of a head-mounted display (HMD). That is, the user is not brought to a virtual world, but the 3D content is shown in the real world, avoiding cybersickness and the size and distance underestimations typical when using stereoscopic displays, while also avoiding user instrumentation.

These PBDs allow the creation of 3D visualizations by freely moving a particle in 3D space at such a high speed (e.g., ∼8 m/s) that visual content is revealed using the persistence of vision (POV) effect (Hardy, 1920), i.e., when an image is perceived as a whole by the human eye due to rapid movement succession (see Figure 1a). Particularly, the class of PBDs that uses acoustophoresis (Hirayama et al., 2019; Martinez Plasencia et al., 2020) is able to deliver visual stimuli that can be felt and heard simultaneously (spatially overlapping). For this reason, this technology is called a multimodal acoustic trap display (MATD) (see Figure 1a).

To produce an image that exists in real 3D space, the MATD uses sound waves (emitted from an array of speakers) to trap a lightweight particle (a polystyrene bead) in free space, which is called acoustic levitation. The position of this particle is updated at a very high update rate so that the POV effect occurs, and the observer perceives it as a full object. Since the particle is updated at such a high speed, the display can create audio (any sound that you could play with a traditional speaker) and tactile feedback (a gentle sensation of touch coming from the display) simultaneously. Since this new volumetric display technology offers multisensory stimulation, it could enable the study of multisensory integration beyond pairs of senses (e.g., visual, auditory, and haptic tasks), as it offers the flexibility to deliver and precisely control visual content alongside tactile stimuli and sound within the same setup. Therefore, this technology could be used in studies exploring multisensory distractor processing, where sensory targets and distractors often need to be placed and presented from the same location (Merz et al., 2019).

The spatio-temporal features of these displays can be considered for possible experimental design around multisensory integration in future studies, replacing 2D screens or HMDs. For instance, the MATD proposed by Hirayama et al. (2019) manages two types of refresh rate, one for particle position and one for rendered images. The particle position refresh rate is ∼10 kHz, taking ∼0.1 ms to update the position of the particle in 3D space. Each image rendered with the MATD is composed from several updates of a single particle. The image refresh rate is ∼10 Hz, taking ∼100 ms for a 3D image to be fully rendered. The particles that this display can levitate and accelerate can have a maximum diameter of ∼2mm and a minimum diameter of ∼1 mm. The size of the images rendered is ∼10 cm^3^, with a maximum velocity of ∼8 m/s. For instance, a sphere of 2-cm in diameter takes ∼100 ms to be fully rendered using a single particle. When an image is rendered at ≤100 ms, it is considered POV time, i.e., when a single moving object along a trajectory is perceived as a whole image and the human eye can see it without flickering.

Similar volumetric displays use the principles of acoustic levitation, although they do not quickly update the particles to render an image (using POV). Instead, in real space, they levitate particles attached to a piece of fabric onto which an image is projected to created levitating displays. Recent work in HCI has shown that these levitating displays enable a good control for interactive presentations (Morales et al., 2019; Morales González et al., 2020).

Other novel techniques that can offer benefits for visual stimulus modulation, particularly for visuo–tactile tasks, include retargeting techniques in mixed reality. These techniques deform the visual space (conflicting an observer’s sense of vision and touch) without the user noticing, thus creating different illusions that can modulate the reliability of visual and tactile interactions. For example, many studies on visuo–haptic integration are limited to haptic modulation through force feedback (using physical devices or motor actuators). Retargeting techniques instead can modulate the perception of touch by exploiting the dominance of the visual system (visual capture; Rock and Victor, 1964), reducing the use of physical haptic devices. They can, for instance, modulate the perception of the quantity of objects (Azmandian et al., 2016), of an object’s weight (Rietzler et al., 2018; Samad et al., 2019), of different textures (Cheng et al., 2017), or of different geometries (Zhao and Follmer, 2018) using limited physical elements (no motors or robots) and relying mainly on visual cues.

In other words, emerging visual image processing and mixed reality technology can enable the study of visuo–haptic integration by reducing the use of physical proxies (e.g., deformable surfaces; Drewing et al., 2009; Cellini et al., 2013), which can be inflexible and more complex to control. Instead, these novel techniques deform the visual space which can be more easily controlled by taking advantage of the visual capture, which is particularly present in spatial tasks (Kitagawa and Ichihara, 2002). Using translational gains, these techniques can even be extended to modulate visual perception involving more complex actions (beyond hand–object interaction in desktop-based experiments), such as walking (Razzaque et al., 2001). Some examples include techniques that modulate the perception of walking speed (Montano-Murillo et al., 2017), walking elevation (Nagao et al., 2017), and distance travelled (Sun et al., 2018). These technologies could open up opportunities to expand and facilitate the study of the integration between vision and proprioception (Van Beers et al., 1999) or between visual and vestibular stimuli (Gu et al., 2006), as well as extend the study of the body schema, which is usually studied for hand interactions (Maravita et al., 2003).

As retargeting techniques mainly employ HMDs to show visual content, other multisensory technologies can easily be combined, for example, headphones to present auditory stimuli, haptic devices, such as vibrational attachments (controllers, suits), and smell delivery devices (external or wearable), as have previously been used in VR settings [e.g., in the work by Ranasinghe et al. (2018)].

### Auditory Stimulation Beyond Headphones

Studies exploring auditory integration commonly modulate sensory information by changing the frequency or synchrony of auditory cues in identification or speech recognition tasks, requiring audio–visual simultaneity (Fujisaki et al., 2004). In these experiments, auditory stimulus delivery is limited to the use of headphones and static sources of sound (speakers). To avoid extra confounding factors, noise control or canceling is also required. However, recent advances in sound manipulation offer new opportunities to deliver and control sound, enabling, for instance, the presentation of directional sound without wearing headphones in a controlled manner. These technological advances could not only help overcome existing limitations but also open up new experimental designs for multisensory integration studies.

Researchers in areas, such as physics, engineering, computer science, and HCI are working on new concepts of controlling sound using ultrasonic manipulation and acoustic metamaterials (Norasikin et al., 2018; Prat-Camps et al., 2020), moving towards the ability of controlling sound just like we do with light (Memoli et al., 2019b). Advances in optics enable the modulation of users’ visual perception through the use of filters and lenses (e.g., cameras and VR headsets). Nevertheless, for sound, this is more challenging, but researchers have already created acoustic lenses to control, filter, and manipulate sound. These techniques are possible thanks to ultrasound phased arrays integrated with acoustic lenses (also called metamaterials) that direct the sound by using acoustic bricks (Memoli et al., 2017). For example, in a theater, a spotlight can be delivered to a single person while others around are in the darkness. But, imagine that a spot of sound is delivered to a single person in the audience while others around that person cannot perceive it. In another example, in a cinema, the movie audio could be played in different languages and delivered to specific persons in the audience (Memoli et al., 2019a). Figure 1b is a simplified representation of sound “bending” around an object by Norasikin et al. (2018). This technique directs sound waves to avoid obstacles (represented by the dashed line in Figure 1b). At the same time, the directed sound is able to not only levitate a small bead above an object but also produce a tactile sensation above the bead in the user’s finger.

The aforesaid sound manipulation can benefit the study of multisensory integration in different ways. Since the direction of sound can be controlled with these acoustic lenses, it is possible to modulate the perceived position of the sound source, even when it is static (Graham et al., 2019). This technology could then be integrated into classical paradigms used in multisensory integration studies, such as temporal/spatial ventriloquism (Vroomen and de Gelder, 2004) and other experimental paradigms studying spatial localization and sound source location using multisensory signals (Battaglia et al., 2003).

Other benefits include the possibility to avoid instrumentation, i.e., avoiding the use of headphones for noise canceling; the ability to precisely modulate the perception of sound location, direction, and intensity inside a room in spatial and temporal tasks, even when the sound source is static; or the possibility to have multiple subjects in an experimental room while auditory stimuli are delivered individually and without causing distractions. Furthermore, through the use of body tracking sensors, this technology could identify a moving person in order to deliver an auditory stimulus while they are walking (Rajguru et al., 2019), which could be suitable for navigation and spatial localization tasks (Rajguru et al., 2020). This opens up opportunities for studies beyond desktop-based experiments and therefore allowing navigation tasks that combine body movement signals, such as auditory–vestibular integration.

Additionally, some of these devices allow multimodal delivery, enabling interactions beyond pairs of senses. For example, the methods by Jackowski-Ashley et al. (2017) and Norasikin et al. (2018) allow the integration with touch (i.e., mid-air tactile stimulation), while the approach by Norasikin et al. (2019) allows visual stimulation *via* reconfigurable mid-air displays. This technology controls directional sound while at the same time producing a haptic sensation on the skin due to the specific frequency of the emitted sound waves. This combination of signals can allow us to present sound and haptic sensations from the same location, which could offer benefits in the study of haptic–auditory integration studies (Petrini et al., 2012).

Moreover, directional sound can be achieved with more traditional speakers (i.e., not involving ultrasound) using the principles of spatial sound reproduction, making it possible to “touch” the sound and interact with it (Müller et al., 2014) and enabling the study of audio-tactile interactions. This technology could be integrated into classical experimental paradigms involving audio–tactile judgments; for example, the audio–tactile loudness illusion (Yau et al., 2010) and other combinations of signals, such as tactile stimulation and music (Kuchenbuch et al., 2014).

While much of this research is still at an early stage (i.e., laboratory explorations), it already points to future opportunities in real environments with promising benefits for expanding research beyond the development of the technology itself and towards its use in psychology and neuroscience research.

### From Physical to Contactless Tactile Stimulation

Much research on touch, in the context of multisensory integration, has focused on visuo–haptic integration (Stein, 2012), although several studies also focus on the integration between haptics and audio (Petrini et al., 2012) and smell (Castiello et al., 2006; Dematte et al., 2006). In most cases, however, haptic information is modulated in size estimation or identification tasks. Haptic sensation is usually modulated through deformable surfaces (Drewing et al., 2009), force feedback (Ernst and Banks, 2002), data gloves (Ma and Hommel, 2015; Schwind et al., 2019), or vibration actuators on skin (Maselli et al., 2016).

These studies rely on tangible objects, so therefore findings on haptic integration with other senses have so far been based on physical touch achieved by using either mechanical actuators or user instrumentation. However, with the accelerated digitization of human experiences produced by social distance restrictions, we see increasing contactless and remote interactions not only in light of the COVID-19 pandemic but also in light of the proliferation of mid-air interactions (Rakkolainen et al., 2020) and the digitalization of the senses (Velasco and Obrist, 2020).

Mid-air interactions allow subjects to control objects from a distance by means of hand gestures and without the need of physical contact. To provide a tactile sensation in mid-air, ultrasonic phased arrays composed of several speakers (see Figure 1c) can be computer-controlled to emit focused ultrasound over distance (e.g., 20 cm) and enable a person to perceive tactile sensations in mid-air without the need of physical attachments, such as a glove. These tactile sensations can be single or multiple focal points on the hand, 3D shapes (Carter et al., 2013), or textures (Beattie et al., 2020). This unique combination is enabling novel interaction paradigms previously only seen in science fiction movies. For example, it is now possible to touch holograms (Kervegant et al., 2017; Frish et al., 2019), as well as levitate objects (Marzo et al., 2015), and interact with them (Freeman et al., 2018; Martinez Plasencia et al., 2020). We can now interact with computers, digital objects, and other people in immersive 3D environments in which we cannot only see and hear but can also touch and feel. This technology is also able to convey information (Paneva et al., 2020) with a huge potential for mediating and studying emotions (Obrist et al., 2015). Furthermore, it has become wearable (Sand et al., 2015) and part of daily-life activities, suggesting a promising potential for dynamic and more natural scenarios, such as online shopping (Kim et al., 2019; Petit et al., 2019), in-vehicle interactions (Large et al., 2019), and home environments (Van den Bogaert et al., 2019), where people can naturally integrate sensory information during daily tasks.

Despite the rapid development of mid-air technologies, efforts to study haptic integration are uniquely directed to physical touch to date, and it is therefore unknown how mid-air touch is integrated with the other senses. For instance, we do not know if the integration of vision, audio, or smell with mid-air touch is similar to what has been found with actual touch, as there are many factors that make physical and mid-air touch different (e.g., physical limits, force, ergonomics, instrumentation, etc.). Here, we see an opportunity to expand the knowledge around mid-air interactions by applying the principles of multisensory integration from the area of psychology and neuroscience. Bridging this gap could open up a wide range of new studies exploring the integration of multiple senses with mid-air touch using the technology recently developed in HCI and further taking advantage of the current knowledge generated in this area. For example, a number of studies have already provided insights that improve our understanding of mid-air haptic stimuli perception in terms of perceived strength (Frier et al., 2019), geometry identification (Hajas et al., 2020b), and tactile congruency (Pittera et al., 2019b), providing compelling evidence of the capability of mid-air haptics to convey information (Hajas et al., 2020a; Paneva et al., 2020).

Recent studies have used mid-air haptics to replicate traditional paradigms used in sensory experiments, such as the rubber hand illusion (Pittera et al., 2019b) and the apparent tactile motion effect (Pittera et al., 2019a). This suggests promising opportunities to use mid-air touch in other tasks involving visuo–tactile judgments, such as the cutaneous rabbit illusion (Geldard and Sherrick, 1972). In the future, it may even be possible to apply mid-air touch to tasks more complex than cutaneous sensations, such as force judgments (e.g., the force matching paradigm; Kilteni and Ehrsson, 2017).

Additionally, mid-air technology can be flexible enough to allow for multisensory experiences. Ultrasonic phased arrays, such as those developed by Hirayama et al. (2019), Shakeri et al. (2019), and Martinez Plasencia et al. (2020) combine mid-air tactile and auditory stimulation simultaneously. They employ speakers emitting sound waves that, at specific frequencies, can be both heard and felt on the skin. In particular, the methods introduced by Jackowski-Ashley et al. (2017) and Norasikin et al. (2018), not only deliver haptics but also parametric audio (i.e., allowing continuous control over every parameter) that can be directed by using acoustic metamaterials. Mid-air haptics has also been largely integrated with visual stimulus presentation *via* virtual and augmented reality (Koutsabasis and Vogiatzidakis, 2019) and multimedia interactions (Ablart et al., 2017; Vi et al., 2017a).

While most of the technology described above is still in the development phase, some devices able to provide mid-air haptics are commercially available. For example, STRATOS Explore and STRATOS Inspire are haptics development kits introduced by Ultraleap^3^ that are are currently available in the market.

### Emerging Smell Technologies and Olfactory Interfaces

The sense of smell is powerful, and research shows that the human nose has similar abilities to those of many animals (Porter et al., 2007; Gilbert, 2008). Therefore, the sense of smell has gained increasing attention in augmenting audio–visual experiences (Spence, 2020). Some studies have explored the integration of smell with vision (Gottfried and Dolan, 2003; Forscher and Li, 2012), audition (Seo and Hummel, 2011), and taste (Dalton et al., 2000; Small and Prescott, 2005). Common strategies to modulate and deliver olfactory cues are based on analog methods, such as smelling scented pens and jars of essential oils (Hummel et al., 1997; Stewart et al., 2010) that are limited by poor control over the scent stimulus delivery. More sophisticated clinical computer-controlled olfactometers have been employed but can often be bulky, static, and noisy (Pfeiffer et al., 2005; Spence, 2012).

Novel olfactory interfaces developed by researchers in the field of HCI can overcome some of those challenges. For example, today smell delivery technology, in addition to being precise and controllable (Maggioni et al., 2019), has become wearable (Yamada et al., 2006), small (Risso et al., 2018), and even fashionable (Amores and Maes, 2017; Wang et al., 2020; see Figure 1c). This portability can facilitate scent delivery in daily activities outside laboratory settings (e.g., home, work), offering opportunities to study smell stimulation in various contexts, such as longitudinal and field studies. For example, attention ability might differ between lab studies and daily-life settings, which can affect the study results (Park and George, 2018). Researchers studying the behavior of the sensory system during daily-life activities (e.g., Sloboda et al., 2001; Low, 2006) might benefit from wearable and miniaturized devices that can be easily carried. Some of these devices not only deliver sensory stimuli on demand but also record data that can be stored in a smartphone for further processing and analysis (Amores and Maes, 2017; Amores et al., 2018).

Furthermore, since wearable scent delivery systems are small and portable, they can easily be integrated with additional multisensory technology and other actuators. For example, Brooks et al. (2020) used a wearable smell delivery device attached to a VR headset to show visual stimuli as well. Moreover, Ranasinghe et al. (2018) added sensory stimulation, such as wind and thermal feedback to provide a multisensory experience and thus induce a sense of presence. In another example, Amores and Maes (2017) and Amores et al. (2018) developed a wearable smell delivery system in the form of a necklace that can be combined with a VR headset and sensors to collect physiological data (e.g., EEG, heart rate), suggesting opportunities for using it while sleeping.

One interesting exploration of emerging wearable smell delivery systems is how to deliver scent stimuli which are released based on physiological data from the body, including moods and emotions (Tillotson and Andre, 2002), brain activity, or respiration (Amores et al., 2018). These new olfactory devices make use of advances in sensors (e.g., biometric and wearable sensors) and enable thinking beyond the constraints of unisensory stimulation. For example, wearable scent delivery systems have been used to modulate the perception of temperature (Brooks et al., 2020), which can enable the study of multisensory integration involving olfactory and somatosensory signals (de la Zerda et al., 2020).

Based on the same principles of directed sound, ultrasound can also be used to control and direct scent stimuli (Hasegawa et al., 2018). Current air-based scent delivery devices such as those employing compressed air (Dmitrenko et al., 2017), fans (Hirota et al., 2013) and vortexes (Nakaizumi et al., 2006), allow great control over the temporal and spatial diffusion of scents (Maggioni et al., 2020). However, these air-based scent transportation systems produce a turbulent flow that disperses the scents with distance decreasing their intensity. Sound-based smell delivery instead uses acoustic beams that produce more laminar scent flow, suggesting promising additional control over the spatial distribution of scents particularly, thus increasing their intensity.

While these efforts are still in the early exploration stage, they again illustrates how technological advances can enable experimental studies to help advance our understanding of multisensory integration. While it may seem far-fetched and beyond current everyday life experiences, wearable and body-responsive technology (e.g., a device that releases a scent based on my heart rate) is in line with growing efforts to design and develop technology that promotes a paradigm shift from human–computer interaction to human–computer integration (Mueller et al., 2020)—a future in which technology becomes part of us (e.g., wearing a device that becomes part of my body and responds based on my body’s signals). As prior research has shown, the sense of one’s own body is highly plastic, with representations of body structure and size particularly sensitive to multisensory influences (Longo and Haggard, 2012). We are seeing initial efforts, sometimes from an artistic design perspective, to explore smell-based emotionally responsive wearable technology. For example, smell has been shown to influence how we feel about ourselves (Tillotson, 2017; Amores et al., 2018), affect our body image perception (BIP) (Brianza et al., 2019) and support sleep and dreaming (Carr et al., 2020). More opportunities around smell can be studied with respect to human sensory perception and integration due to these ongoing technological advances.

### Emerging Gustatory Technologies and Interfaces

Unlike other sensory modalities that can be stimulated externally (e.g., vision, audio, and smell), taste stimulation occurs inside the body, and this can be more complex and invasive. A common area of study is around odor–taste integration (Dalton et al., 2000) given the multisensory nature of flavor perception (Prescott, 2015). However, since food perception is more broadly considered one of the most multisensory experiences in people’s everyday lives (Spence, 2012), different studies have also explored the integration of taste with vision (Ohla et al., 2012), audition (Yan and Dando, 2015), and touch (Humbert and Joel, 2012). In most cases, however, gustatory cues have been modulated by changing the concentration of taste stimuli in reaction and detection tasks (Overbosch and De Jong, 1989). For such tasks, precision is crucial, and while some studies use simple methods, such as glass bottles (Pfeiffer et al., 2005), precise taste control stimulation can be achieved through well-established gustatometers consisting of either chemical or electric stimulation (Ranasinghe and Do, 2016; Andersen et al., 2019). However, controlling taste delivery through these methods can be unnaturalistic and is constrained-to-in-lab settings.

Novel interfaces from HCI may represent more naturalistic interactions and enable new contexts for studying the multisensory integration of taste with other senses. For example, mixed reality^4^ systems are also employed to modulate the perception of taste in augmented (Narumi et al., 2011b; Nishizawa et al., 2016) and virtual reality (Huang et al., 2019) suitable for visuo–gustatory interactions in wearable settings. Recent systems have also enabled the combination of multiple senses, for example, involving vision, olfaction, and gustation (Narumi et al., 2011a), which can facilitate studying integration beyond pairs of senses. These systems alter the visual attributes (e.g., color) of a seen physical item (e.g., cookie, tea) by means of image processing to vary its flavor perception.

Meanwhile, emerging tongue-mounted interfaces (Ranasinghe et al., 2012) do not use physical edible items but are able to produce, to a certain degree, sour, salty, bitter, and sweet sensations by electric and thermal stimulation without using chemical solutions, promising to be user-friendly (Ranasinghe and Do, 2016). These interfaces can be combined with other sensory modalities as well, such as smell and vision, using common objects for a more natural interaction, such as drinking a cocktail (Ranasinghe et al., 2017).

In another example, touch-related devices have enabled the study of taste perception by varying weight sensations (Hirose et al., 2015), biting force (Iwata et al., 2004), or vibrotactile stimuli (Tsutsui et al., 2016) suitable for studying a combination of gustatory and proprioceptive signals. Precise control of taste stimuli quantities can also be achieved through novel food 3D printing techniques (Khot et al., 2017; Lin et al., 2020), which permit the design and creation of physical food structures with controllable printing parameters, such as infill pattern and infill density (Lin et al., 2020). This control capability could be suitable for customizing and equalizing conditions during multisensory integration experiments; for example, giving the same concentration of taste stimuli across subjects while enabling a more natural taste stimulation (e.g., an actual cookie or chocolate treat), unlike using electrical stimulation (Spence et al., 2017), which can be invasive. Many other examples can be seen in the field of HCI for enhancing and modulating taste perception *via* different senses [e.g., see the work by Velasco et al. (2018) for a review of multisensory technology for flavor augmentation].

An emerging approach based on the principles of acoustic levitation is computer-controllable levitating food (Vi et al., 2017b). This technology consists of a contactless food delivery system able to deliver food morsels to the user’s tongue without the need of pipettes or electrodes. This contactless interaction can be suitable for delivering taste stimuli while maintaining a sterile and clean environment. Unlike electrical stimulation, levitating food techniques offer the possibility to deliver actual food, i.e., multiple morsels simultaneously in 3D, enabling the manipulation of the food’s trajectories. This technology has been extended to synchronized integration of levitated food with visual, olfactory, auditory, and tactile stimuli (Vi et al., 2020), enabling systematic investigations of multisensory signals around levitated food and eating experiences. For example, with this system, Vi et al. (2020) found that perceived intensity, pleasantness, and satisfaction regarding levitating taste stimuli are influenced by different lighting and smell conditions. This approach thus opens up experimentations into new tasting experiences (e.g., molecular gastronomy; Barham et al., 2010).

The aforesaid new approach can extend the study of multisensory integration in several ways. For example, studies exploring olfactory–gustatory integration can benefit from the multimodal functionalities of this technology. Different mixtures could be created in mid-air by levitating different droplets of different solutions with precision, allowing researchers to dynamically change experimental conditions (e.g., different tastes) while at the same time controlling smell stimulus presentation in terms of time (precise control of delivery duration) and location (directional delivery toward the subject’s nose). Additionally, since levitated food does not involve physical actuators, this could facilitate its implementation within VR environments (e.g., in visuo–gustatory interactions), avoiding the need to track additional elements (e.g., the subject’s hands, spoons) (Arnold et al., 2018). Finally, levitating food approaches can also facilitate the study of multisensory spatial interactions, given that food stimuli can be delivered to the subject’s mouth from different locations.

The multimodal properties of these new gustatory technologies and interfaces can be applied to classical paradigms used in the study of multisensory integration, for example, in studies involving gustatory and olfactory interactions, such as odor–taste learning (Small and Green, 2012) or involving gustatory and auditory interactions, such as the sonic chip paradigm (Spence, 2015). Overall, the technology described in this section is opening up a wide range of opportunities not only in multisensory integration research but also in the context of eating and human–food interaction (Velasco and Obrist, 2021).

## Discussion, Conclusion, and Future Research

The aim of this review was to reflect upon the opportunities that advances in multisensory technology can provide for the study of multisensory integration. We have exemplified how researchers in the field of multisensory integration could derive inspiration and benefit from novel emerging technologies for visual, auditory, tactile, and also olfactory and gustatory stimulation. Apart from describing the level of control that new interfaces and devices offer, we have highlighted some of the new flexibility such technologies provide, such as how the different senses can be stimulated simultaneously and how the study of multisensory integration can be moved beyond the laboratory into more naturalistic and newly created settings, including physical/real and digital/virtual worlds.

While multisensory technology is advancing and revealing new opportunities for the study of multisensory integration, a major issue we would like to highlight is how responsibility is shared between humans and technology. Computing systems today have become ubiquitous and increasingly digital. An example of this is the evolution from human–computer interaction—a stimulus–response interplay between humans and technology (Hornbæk and Oulasvirta, 2017)—towards human–computer integration (Mueller et al., 2020)—a symbiosis in which humans and software act with autonomy (Farooq and Grudin, 2016). For example, multisensory technology becomes more connected to our body, emotions, and actions since sensors can be worn that allow mobile interactions (Wang et al., 2020). Responses from systems are mediated by the user’s biological responses and emotional states (Amores and Maes, 2017). Virtual environments allow one to embody virtual avatars, thus creating the feeling of body ownership and the sense of presence (i.e., the feeling of being there), with realistic environments no longer limited to audio–visual experiences but also including touch (Sand et al., 2015), smell (Ranasinghe et al., 2018), and taste experiences (Narumi et al., 2011b).

This increased symbiosis between humans and technology (Cross and Ramsey, 2020) leads to the challenge of a shared “agency” between humans and digital systems. Agency or, more precisely, the sense of agency (SoA) is crucial in our interaction with technology and refers to the feeling of “I did that” as opposed to “the system did that,” supporting a feeling of being in control (Haggard, 2017). The SoA arises through a combination of internal motoric signals and sensory evidence about our own actions and their effects (Moore et al., 2009). Therefore, increasing sensory evidence by giving the subjects multisensory cues during interactions can make technology users more aware of their actions and the consequences of these, thus promoting a feeling of responsibility (Haggard and Tsakiris, 2009). Since recent technology posits the user in environments that are not fully real (e.g., virtual or augmented) and where users’ actions are sometimes influenced (e.g., autocompletion predictors) or even automated (e.g., autonomous driving), multisensory signals can help the users to feel agency during the interaction with technology, even though they are not the agent of the action (Banakou and Slater, 2014). Emerging research is examining how to improve the SoA during human–computer interaction, for example, by exploring motor actuation without diminishing the SoA (Kasahara et al., 2019), exploring appropriate levels of automation (Berberian et al., 2012), or exploring how the SoA can be improved through olfactory interfaces (Cornelio et al., 2020). Despite such efforts, it has been suggested that “the cognitive coupling between human and machine remains difficult to achieve” (Berberian, 2019), so therefore further research is needed. However, in light of this review, we argue that, in a digital world in which users can see, hear, smell, touch, and taste just like they do it in the real world, it can provide the sensory signals that they need to self-attribute events, thus facilitating the agency delegation between humans and systems.

In summary, we believe that the SoA is a key concept that may become increasingly important to consider in the study of multisensory integration especially when moving from laboratory to real-world environments. Despite the astonishing technological progress, it is worth acknowledging that some of the technologies—interfaces and devices—described in this review are still in the development phase, and although their principles are possible in theory and often demonstrated in proofs-of-concepts, more testing is needed. Additionally, some of the devices discussed in our review lack studies with human participants. For example, the volumetric displays illustrated in Figure 1c have only been tested in laboratory settings with no further exploration of areas in which they could be useful (e.g., psychophysics studies). This highlights the main motivation underlying our review – to make researchers aware of these emerging technological opportunities for studying multisensory integration. While technological feasibility has been demonstrated, there is a lack of understanding of how these new devices can benefit the study of human sensory systems. We hope that this review sparks interest and curiosity among those working in other fields and opens up mutually beneficial research avenues to advance both engineering and computing and our understanding of the human sensory systems. Indeed, we believe that strengthening the collaborations between psychology, neuroscience, and HCI, maybe prove to be fruitful for the study of multisensory integration.

Bringing these disciplines closer together may benefit the study of multisensory integration in a reciprocal fashion, that is, new technologies can easily be adapted to classical experimental paradigms used in neuroscience research. Similarly, principles and theories emerging from neuroscience research that have provided evidence of how the human sensory system works can be used to develop new technologies, contributing to a more accurate human–computer integration symbiosis.

## Author Contributions

All authors listed have made a substantial, direct and intellectual contribution to the work, and approved it for publication.

## Conflict of Interest

The authors declare that the research was conducted in the absence of any commercial or financial relationships that could be construed as a potential conflict of interest.

## Footnotes

1 We refer to human–computer interaction as a stimulus–response interplay between humans and any computer technology that can go beyond common devices, such as PC or laptops (Dix et al., 2004).

2 We refer to human–computer integration to a partnership between humans and any computer technology that can go beyond common devices, such as PC or laptops (Farooq and Grudin, 2016).

3 https://www.ultraleap.com/haptics/

4 “Mixed reality is the merging of real and virtual worlds to produce newenvironments and visualizations, where physical and digital objects co-exist and interact in real time.” (Milgram and Kishino, 1994).
